# Development of an online tool for sodium intake assessment in Mexico

**DOI:** 10.26633/RPSP.2019.90

**Published:** 2019-12-06

**Authors:** Eloisa Colín-Ramírez, Raúl Cartas-Rosado, Paola Vannesa Miranda Alatriste, Ángeles Espinosa Cuevas, JoAnne Arcand, Josefina C Morales Guerrero, Lorena Cassis Nosthas, Susana Rivera-Mancía, Maite Vallejo Allende, Ricardo Correa-Rotter

**Affiliations:** 1 Cátedras CONACYT— Instituto Nacional de Cardiología Ignacio Chávez Mexico City Mexico Cátedras CONACYT— Instituto Nacional de Cardiología Ignacio Chávez, Mexico City, Mexico.; 2 Electromechanical Instrumentation Department Instituto Nacional de Cardiología Ignacio Chávez Mexico City Mexico Electromechanical Instrumentation Department, Instituto Nacional de Cardiología Ignacio Chávez, Mexico City, Mexico.; 3 Department of Nephrology and Mineral Metabolism Instituto Nacional de Ciencias Médicas y Nutrición Salvador Zubirán Mexico City Mexico Department of Nephrology and Mineral Metabolism, Instituto Nacional de Ciencias Médicas y Nutrición Salvador Zubirán, Mexico City, Mexico.; 4 Faculty of Health Sciences University of Ontario Institute of Technology Oshawa, Ontario Canada Faculty of Health Sciences, University of Ontario Institute of Technology, Oshawa, Ontario,Canada.; 5 Department of Food Science and Technology Instituto Nacional de Ciencias Médicas y Nutrición Salvador Zubirán Mexico City Mexico Department of Food Science and Technology, Instituto Nacional de Ciencias Médicas y Nutrición Salvador Zubirán, Mexico City, Mexico.; 6 Sociomedical Research Department Instituto Nacional de Cardiología Ignacio Chávez Mexico City Mexico Sociomedical Research Department, Instituto Nacional de Cardiología Ignacio Chávez, Mexico City, Mexico.

**Keywords:** Dietary sodium, diet, sodium chloride, biomedical technology, Mexico, Sodio en la dieta, cloruro de sodio, tecnología biomédica, México, Sódio na dieta, cloreto de sódio, tecnologia biomédica, México

## Abstract

Excess sodium intake is associated with adverse health effects, and reducing its intake is a strategy that improves population health. However, estimating sodium intake is challenging and new options for assessment are needed. This review describes the design and development of a web-based, publicly-accessible, dietary sodium intake screening tool (*Calculadora de Sodio*) for individuals in Mexico. Sodium data from 2017 – 2018 for 3 429 packaged foods, 655 restaurant and cafeteria foods, and 320 home-style meals and street foods (determined by chemical analysis) comprised the 71-question tool. It was piloted with 10 nutrition experts for feedback on content and face validity; and with 30 potential users to test its usability and interface. Improvements were made to content, language, and formatting following the pilot. Its predictive validity will be established in the future. The *Calculadora de Sodio* provides instant feedback on an individual’s average daily sodium intake, computed by frequency of intake, average number of servings, and sodium content per serving of each sodium-focused food category. This is the first web-based dietary sodium screening tool developed for the general population of Mexico. It is an efficient and practical way to assess sodium intake and can serve as a model for similar tools for other countries and regions.

## RESUMEN

La ingesta excesiva de sodio se asocia a efectos nocivos para la salud y su reducción constituye una estrategia para mejorar la salud de la población. Sin embargo, es complicado estimar la ingesta de sodio y se necesitan nuevas alternativas para evaluarla. En este examen se describe el diseño y la creación de una herramienta en línea y de acceso público con el fin de establecer la ingesta de sodio en la alimentación (denominada la “calculadora de sodio”) para la población de México. La herramienta, consistente en 71 preguntas, incluye los datos de sodio correspondientes al 2017-2018 de 3 429 alimentos envasados, 655 alimentos de restaurantes y cafeterías y 320 comidas caseras y alimentos de puestos de venta de la calle (determinados mediante análisis químicos). Se hizo una prueba piloto con diez nutricionistas que aportaron su opinión experta en materia de validez del contenido y diseño, además de 30 usuarios potenciales que probaron la facilidad de uso y su interfaz. Tras la prueba piloto, se incluyeron mejoras de contenido, idioma y formato. En el futuro se podrá determinar su valor predictivo. La calculadora de sodio ofrece una evaluación instantánea sobre la ingesta de sodio promedio diaria de una persona, calculada según la frecuencia de la ingesta, la cantidad promedio de raciones y el contenido de sodio por ración de cada categoría de alimentos con sodio. Esta es la primera herramienta en línea de detección de sodio en los alimentos creada para la población general de México. Es una manera eficaz y práctica de evaluar la ingesta de sodio, y puede servir de modelo para herramientas similares en otros países y regiones.

## RESUMO

A ingestão de sódio em excesso está associada a efeitos adversos à saúde, e a redução do consumo alimentar de sódio é uma estratégia que contribui para a melhoria da saúde das pessoas. Porém, como é difícil estimar a ingestão de sódio, são necessários novos métodos de avaliação. Neste estudo são apresentados o projeto e o desenvolvimento de um instrumento on-line e aberto ao público (denominado ‘’calculadora de sódio*’’*) para a triagem da ingestão alimentar de sódio por indivíduos no México. O instrumento contém 71 perguntas preparadas com base em dados do teor de sódio, coletados no período de 2017 a 2018, de 3.429 alimentos embalados, 655 alimentos comercializados em restaurantes e lanchonetes e 320 refeições do tipo caseiro e comidas de rua (medidos com análises químicas). Um teste-piloto foi realizado com 10 especialistas em nutrição, que fizeram observações sobre a validade de conteúdo e a validade aparente do instrumento, e 30 possíveis usuários que avaliaram sua usabilidade e interface. O conteúdo, os enunciados e o formato foram aperfeiçoados após o teste-piloto. A validade preditiva do instrumento será determinada futuramente. A ‘’calculadora de sódio’’ proporciona uma avaliação imediata da ingestão alimentar média de sódio de uma pessoa, calculada pela frequência de consumo, número médio de porções e teor de sódio por porção de cada categoria de alimento que contém sódio. Este é o primeiro instrumento on-line para a triagem de sódio alimentar desenvolvido para a população do México. É um recurso eficiente e prático para avaliar a ingestão de sódio e pode servir de modelo para o desenvolvimento de instrumentos semelhantes em outros países e regiões.

Excess sodium intake is associated with adverse health effects such as elevated blood pressure. A recent systematic review of eight high-quality studies on salt and health outcomes found adverse effects reported by five studies; benefits of sodium restriction on blood pressure reported by one; and neutral results reported by two (1). In 2017, high sodium intake accounted for 3.2 million deaths globally (2). To reduce blood pressure and cardiovascular risk at the population level, the World Health Organization (WHO) now recommends < 2 000 mg of sodium daily (3).

In 2017, dietary sodium intake—measured by 24 hr urinary sodium excretion (gold standard)—among a cohort of adults in Mexico City was estimated at 3 150 mg/day, about 3 735 mg in men and 2 875 mg in women (4). Another study (5) found that bread products (savory and sweet) were identified as major contributors to daily sodium intake, accounting for 16%, followed by processed meat (8%), natural cheeses (5%), tacos (5%), and breakfast cereals (2%). In many countries, the main source of dietary sodium is processed foods and ready-made meals (3); in Argentina, it was estimated that 65% – 70% of dietary sodium intake comes from processed foods (6).

Despite knowing that processed and prepared foods contribute greatly to the high sodium of western diets, individuals have misconceptions or lack awareness of the sources and quantities of sodium in their diets (7). The complexity of estimating sodium intake outside of the clinical setting may be partly to blame for this gap. Biomarkers and dietary surveys used in a clinic are not easily applied to the general public. These tools require time-consuming techniques, trained personnel, and analysis. Even in the clinical setting, dietary surveys may underestimate sodium intake because estimating its content if many foods is challenging, especially of food eaten away from home.

To address these challenges, we developed a web-based, publicly-accessible screening tool (*Calculadora de Sodio*) to estimate sodium intake using foods included in the Mexican diet. This online tool was designed to provide instant feedback on the user’s sodium intake and its main dietary sources. It was based on a similar tool developed for the Canadian population (8). This paper describes the design and development of the *Calculadora de Sodio*. The project was approved by the Research Review Board of the *Instituto Nacional de Cardiología Ignacio Chávez* (The Ignacio Chávez National Institute of Cardiology).

## DEVELOPING THE ONLINE SODIUM SCREENING TOOL

### Improving on the Dietary Sodium Food Frequency Questionnaire

A previous work by the authors (5) identified 26 food categories that accounted for 52.7% of total sodium consumed per person in an adult cohort in Mexico City. These food categories had been used previously to develop a self-administered, paper-based, high-sodium food frequency questionnaire for the adult population. This questionnaire proved useful for identifying individuals at greater risk of high sodium intake. It considered a total score ≥ 51.2 to be significantly associated with a sodium intake ≥ 3 000 mg/day (Odds Ratio: 3.12; 95%CI: 1.03 – 9.44, *P* = 0.04) after adjusting for sex, age, and body mass index (9).

The paper-based questionnaire served as the starting point for the online Dietary Sodium Food Frequency Questionnaire (DSFFQ). The online version, however, was designed to include additional items for which sodium content data had not been available previously, i.e., restaurant meals, *comida corrida* (home-style meals from local kitchens), street foods, and more. The online version of the DSFFQ included 71 questions organized by sodium-focused food categories: (a) foods and meals prepared outside the home; (b) home-style meals and local street foods; (c) ice cream, desserts, pastry, cakes, breads, and cookies; and (d) other processed or packaged foods. The last category (d) also contained the processed and packaged foods, such as salty condiments, seasonings, ingredients, etc., used to prepare snacks and meals at home. Two questionnaire items specifically inquired about table-salt, i.e., salt used in cooking at home or added at the table.

Every DSFFQ item asks about the frequency of intake (times per day/week/month/year) for each specific food category and the number of pre-defined serving sizes usually consumed. Serving sizes were defined by standard intakes or units (pieces, cups, glasses, spoons, bowls, and portions as served by the food establishment) and were incorporated into each question. When variations in sodium content were found within a particular food type, the DSFFQ did not ask about the low-sodium option or those naturally low in sodium. For example, unseasoned rice, natural oats, tea/black coffee, fresh meat/fish, fresh vegetables were not considered in the calculation of sodium content for the relevant category.

### Estimating sodium content of packaged and restaurant foods

The DSFFQ questions on packaged and restaurant foods used the mean sodium content from a dataset containing sodium information for 3 429 packaged and 655 restaurant and cafeteria foods. Information on packaged foods was collected in 2017 –2018 by reviewing food labels at two retail chains in Mexico City and on the websites of two additional retailers. For restaurant foods, data was collected during the same period from 17 restaurant chains that operate in Mexico City and also report sodium content for all or some of their food: 9 fast-food and take-out restaurants, 3 cafeterias, 4 table-service restaurants, and 1 ice cream shop. Sodium data was collected from the menus at the establishments or from their websites. Seven of the 17 establishments reported sodium content on their United States websites but not on their Mexican counterpart; in these cases, we collected sodium data from the United States website, but only for those items sold in Mexico City.

### Estimating sodium content of home-style and street foods

For food items from local kitchens that serve home-style meals and street foods—both highly consumed among the Mexico City population—sodium content was determined by chemical analysis of inorganic elements. Personnel collecting food samples and performing the chemical analyses had been trained by the Department of Food Science and Technology at the *Instituto Nacional de Ciencias Médicas y Nutrición Salvador Zubirán* (The Salvador Zubirán National Institute for Medical Sciences and Nutrition). Samples of 32 different food products in five municipalities of Mexico City were collected (one at each cardinal point and central point) in May 2017 – April 2018; food samples were collected from two different establishments within each of the five municipalities, for a total of 320 samples collected and analyzed to determine nutritional content. Sodium content was determined per portion and per 100 g. For each of the 32 food products, the mean sodium content was determined with 10 different samples. Sodium data for additional street foods was also collected from the Tables of Composition of Mexican Foods and Food Products (condensed version, 2015, available at https://www.incmnsz.mx/2019/TABLAS_ALIMENTOS.pdf).

## SOFTWARE AND ACCESS

The *Calculadora de Sodio, based on the DSFFQ,* was programmed using Quasar Framework (https://quasar.dev/; SC PULSARDEV SRL, Bucharest, Romania) and is hosted by GitHub at https://calculadorasodio.github.io/. It was programmed to be accessed from a personal computer or mobile device. The link to the *Calculadora de Sodio* is currently available on the website of the Tlalpan 2020 study at http://www.tlalpan2020.mx/. Further dissemination will occur via social media and academic and non-academic events, as well as by incorporating the link onto other relevant health-related webpages.

## FINE-TUNING THE *CALCULADORA DE SODIO*

### Pilot-testing with experts

The *Calculadora de Sodio* was pilot tested with 10 nutrition experts for content and face validity using a Likert scale. Experts were clinical practitioners or nutritional epidemiologists with expertise in dietary intake assessment, dietary sodium reduction, or dietary surveys. They were asked to evaluate the clarity of the instructions and questions, the extensiveness of the online DSFFQ, and the feasibility of applying it to the general adult population. Additionally, the experts were asked if the DSFFQ was missing any questions necessary for estimating habitual daily sodium intake.

Overall, 7 of the 10 experts disagreed/completely disagreed with the statement that the DSFFQ was too extensive regarding the variety of relevant dietary sodium sources. Nine disagreed/completely disagreed with the statement that it was missing any questions on relevant food sources of sodium; however, one expert recommended adding powdered chicken broth to the seasonings category since it is often used to prepare soup and season meals while cooking.

### Pilot-testing with potential users

After the experts’ evaluation, the *Calculadora de Sodio* was pilot tested on 30 adults from the general public who volunteered to provide input. Volunteers were either graduate students working with the research team or a researcher’s family member. They completed the *Calculadora de Sodio* online, and commented, in free form, on the usability of the interface, the clarity of the instructions and questions, and how much time it took to complete. None of the participants complained about the usability of the interface and clarity of instructions and questions, but three asked for clarification on the portion size in one question. The mean time reported to complete the survey was 19.4 ± 5.5 minutes.

### Final version

Content improvements were made following the expert evaluation. Language and formatting modifications were based on both the experts’ and the general users’ comments.

## DIETARY SODIUM REPORT

The *Calculadora de Sodio* provides instant feedback on a user’s estimated average daily sodium intake, calculated by frequency of intake, usual number of servings per day, and the estimated sodium content per serving of each sodium-focused food category. The estimated daily sodium consumed is compared to the WHO recommendation for adults of < 2 000 mg/day (3). This information is included on the report generated upon survey completion, along with the relative percent contribution of each of the four major food categories to the total sodium intake. These categories were rearranged as follows for reporting purposes: (a) foods and meals prepared outside home (including home-style meals and local street foods); (b) ice cream, desserts, pastry, cakes, breads, and cookies; (c) table salt and salty seasonings; and (d) processed or packaged foods.

## DISCUSSION

High intake of sodium is the top dietary risk factor for death and disability among the general public (2). Restricting its intake is one of the most commonly recommended dietary strategies for patients suffering from salt-avid conditions, such as hypertension, heart failure, and chronic kidney disease, among others. Limiting dietary sodium intake is also endorsed by WHO for reducing blood pressure and cardiovascular risk at the population level (3); and more recently, the National Academies of Sciences, Engineering, and Medicine, in their 2019 Dietary Reference (10) set a level of 1 500 mg/day for sodium intake in adults. Thus, evaluating sodium intake in both the clinical and population setting is crucial for the management and prevention of these salt-avid conditions.

Currently, there are several methods available to assess sodium intake, among which the 24 hr-urine collection is the gold standard. Other methods are indirect ways of estimating dietary sodium intake, including multiple-day food records, food recalls, and food frequency questionnaires, which are commonly used despite their limitations. Most of these methods require trained personnel and/or burdensome techniques that are not easily applied outside the clinical setting and do not provide timely feedback to the user.

The web-based dietary sodium screening tool that we developed, *Calculadora de Sodio*, provides immediate and personalized feedback. Plus, it includes new data on the sodium content of common Mexican street foods and home-style meals that are frequently consumed in Mexico City due to their practicality and low price. Indeed, a national survey (11) of 1 000 adult participants revealed that nearly 3 in 10 adults had the afternoon meal (usually 1 pm – 3 pm) outside the home, and of those, 32% preferred tacos, 19% tortas, and 10% *comida corrida* (home-style meals from local kitchens). Thus, adding these foods to the online screening tool may allow more accurate assessment of sodium consumption because it includes the regional foods commonly consumed in our population. Most of this information was not previously available—it was generated by the food sampling protocol of this study.

The *Calculadora de Sodio* is publicly available for use among the general adult population and by clinicians. It is also freely available for the scientific community. Future research will validate this screening tool against 24-hr urinary sodium excretion.

A similar dietary sodium screening tool, the “Sodium Calculator” (www.projectbiglife.ca), was developed for the Canadian population (8) by a member of our group at the University of Ontario Institute of Technology (Ontario, Canada). The Canadian Sodium Calculator contains 23 questions and served as a precedent for the Mexican *Calculadora de Sodio.* The Canadian tool is being validated in the clinical and population settings, and an extended version with > 70 question will soon be publicly available.

Emerging mobile technologies are seen as valuable tools for collecting and assessing dietary intake. They are promising for improving or facilitating dietary assessment in the research setting through more cost-effective, less laborious, and more acceptable ways of data collection (12); however, they need to be continually evaluated for potential improvements and validity.

The *Calculadora de Sodio* is the first tool of its kind for the Mexican population, and although pending validation, it has the potential to be used in the clinical and population settings to assess sodium intake more efficiently. Most importantly, the *Calculadora de Sodio* could allow patients to self-monitor.

### Limitations

The development of this tool had certain limitations. The online DSFFQ, like all self-reporting methods, has an inherent individual bias related to self-reported dietary intake. The sodium content of processed foods and foods eaten away from home may vary greatly. In Mexico, there is limited information on sodium for restaurant foods, especially those in sit-down restaurants—perhaps due to the lack of a national policy on reporting nutritional composition. So, this tool may need to be updated as new sodium data on restaurant meals and packaged foods becomes available. There is also a potential for information bias or underestimation because sodium content was collected from food labels, menus, and websites. Nonetheless, the *Calculadora de Sodio* has the strength of being based on local food sodium data and dietary intake data derived from a population in Mexico City. Moreover, chemical analysis was used to quantify data on sodium content in *comida corrida* and street foods. Additionally, because this is a population-specific tool, it may not be valid for populations with different food availability and local dietary patterns, in Mexico and beyond. Lastly, since this tool is only accessible by Internet or mobile device, it may preclude the least educated and those with the lowest incomes from using it.

### Conclusions and recommendations

The *Calculadora de Sodio* is the first web-based dietary sodium screening tool developed for the Mexican population. This screening tool has the potential to be used in the clinical and population settings as an efficient and practical way to assess sodium intake. Predictive validity will be established in the future.

Although this screening tool is specific to the population of Mexico City, it can be adapted to other settings and may be used as a guide to develop similar tools for other countries or regions. Future versions should be based on local dietary intake and sodium content data to reliably reflect local dietary patterns. Additionally, it is essential to look into options for making this type of tool available to the entire targeted population—including those with limited access to the Internet—so that all can benefit from dietary sodium screening and the health benefits of reducing sodium intake.

## Acknowledgements.

We would like to thank to Arturo Aldair García Romero, student at the Universidad Nacional Autónoma de México, for his valuable work on programming the salt calculator’s webpage.

## Funding.

This work was supported by the National Council of Science and Technology, Mexico, through research grants No. SALUD-2016-C02–272561 and Cátedras CONACYT 1591. The funders had no role in the study design, data collection or analysis, decision to publish, or preparation of the manuscript.

## Author contributions.

ECR, RCaR, and JA conceived the original idea. ECR, PMA, and AEC collected the data. ECR and RCaR analyzed the data. JCMG and LCN planned the experiments and contributed data. AEC, PMA, RCoR, SRM, and MVA interpreted the results; ECR wrote the paper. All authors reviewed the paper and approved the final version.

## Disclaimer.

Authors hold sole responsibility for the views expressed in the manuscript, which may not necessarily reflect the opinion or policy of the *RPSP/PAJPH* and/or PAHO.
